# Whole-genome sequencing of a large collection of *Myroides odoratimimus* and *Myroides odoratus* isolates and antimicrobial susceptibility studies

**DOI:** 10.1038/s41426-018-0061-x

**Published:** 2018-04-04

**Authors:** Florian Gunzer, Wolfram W. Rudolph, Boyke Bunk, Isabel Schober, Sonja Peters, Theres Müller, Boris Oberheitmann, Percy Schröttner

**Affiliations:** 1 Institut für Medizinische Mikrobiologie und Hygiene, Medizinische Fakultät Carl Gustav Carus, TU Dresden, Fetscherstr. 74, 01307 Dresden, Germany; 2 Institut für Virologie, Medizinische Fakultät Carl Gustav Carus, TU Dresden, Fetscherstr. 74, 01307 Dresden, Germany; 3 Leibniz-Institut DSMZ-Deutsche Sammlung für Mikroorganismen und Zellkulturen GmbH, Inhoffenstrasse 7 B, 38124 Braunschweig, Germany; 4 Leibniz-Zentrum für Marine Tropenforschung (ZMT), Fahrenheitstrasse 6, 28359 Bremen, Germany; 5 Q-Bioanalytic GmbH, Fischkai 1, 27572 Bremerhaven, Germany

## Abstract

The genus *Myroides* comprises several species of Gram-negative, non-motile, and non-fermenting bacteria, which have been regarded as non-pathogenic for decades. Multiple recent reports, however, underscore the pathogenic potential that *Myroides* sp. possesses for humans. These bacteria seem to be resistant to a wide range of antibiotics (including ß-lactams and aminoglycosides). Therefore, treatment options are limited. Knowledge of antimicrobial resistance, however, is based on only one meaningful comprehensive study and on data published from case reports. This lack of data motivated us to test 59 strains from our *Myroides* collection (43 *M. odoratimimus* and 16* M. odoratus*) for resistance against 20 commonly used antibiotics. We also performed molecular analyses to reveal whether our bacteria harbor the genus-specific *M. odoratimimus* metallo-ß-lactamase (MUS-1) or the *M. odoratus* metallo ß-lactamase (TUS-1), and other ß-lactamases, which may provide an explanation for the extended antimicrobial resistance.

## Introduction

The bacteria known as *Myroides* today were first described as *Bacterium faecale aromaticum* by Stutzer in 1923^1^. Six years later, in 1929, these bacteria were renamed *Flavobacterium odoratum*^2^. Finally, in 1996, Vancanneyt et al. undertook a new reclassification based on modern techniques that included DNA–rRNA hybridization, DNA–DNA hybridization, analysis of whole-cell protein patterns, fatty acid composition, and the phenotype^3^. Based on their results, they introduced a new genus, *Myroides*, with the species *M. odoratus* (formerly *F. odoratum*) and a new species, *M. odoratimimus*, into the bacterial taxonomy^3^. The bacteria are Gram-negative, non-motile, non-fermenting, and rod-shaped. Their natural habitat is soil and water^3–5^. Infections caused by *Myroides* sp. comprise necrotizing fasciitis, soft tissue infections, ventriculitis, pneumonia, and sepsis. Immunocompromised patients are mostly affected, although being immunocompromised does not seem to be absolutely prerequisite to acquiring a *Myroides* infection^6–12^. In addition, two studies on outbreaks have been published^13,14^. Furthermore, the outcome was fatal in two case reports^9,13–15^. Since *Myroides* sp. are resistant to a wide range of antibiotics, including ß-lactams and aminoglycosides, choosing an appropriate empirical antimicrobial therapy is challenging. Moreover, knowledge about the specific resistance profile of *Myroides* sp. is based on data mainly obtained from case reports and two publications^16,17^. In the present study, we therefore compare minimal inhibitory concentration (MIC) results for 20 important antibiotics against 59 strains from our *Myroides* collection (43 *M. odoratimimus* and 16* M. odoratus*). The data were evaluated according to the guidelines published by EUCAST in 2017. Furthermore, PCR analysis and whole-genome sequencing approaches were applied to detect corresponding genes of important ß-lactamases of the OXA-types, VIM-types, IMP-types, AmpC-types, KPC-types, and NDM-types and for the *Myroides* sp.-specific enzymes MUS-1 and TUS-1.

## Results

### Antimicrobial susceptibility

The susceptibility profile and the MIC distribution of all strains tested are summarized in Tables 1 and 2. The MIC results of each strain are additionally provided in Table S1. Only a few *M. odoratimimus* and *M. odoratus* strains are susceptible to ampicillin (*M. odoratimimus*, *n* = 1; *M. odoratus*, *n* = 5) and piperacillin/tazobactam (*M. odoratimimus*, *n* = 2; *M. odoratus*,* n* = 0). Interestingly, more of the tested strains showed intermediate susceptibility to ampicillin (*M. odoratimimus*, *n* = 19; *M. odoratus*,* n* = 2) than to piperacillin/tazobactam (*M. odoratimimus*, *n* = 9; *M. odoratus, n* = 0). No strain was susceptible to ceftazidime, cefepime, and aztreonam. In total, 4 *M. odoratimimus* strains and no *M. odoratus* strain were susceptible to imipenem. In contrast to this observation, 32 *M. odoratimimus* and 8 *M. odoratus* strains were susceptible to meropenem. The MIC results of the quinolones—ciprofloxacin, levofloxacin, and moxifloxacin—were also compared in this study. Most strains were susceptible to moxifloxacin (*M. odoratimimus*, *n* = 39; *M. odoratus*,* n* = 15). In contrast to this, 5 *M. odoratimimus* strains and 7 *M. odoratus* strains tested were susceptible to levofloxacin. Only 1* M. odoratimimus* strain and 1* M. odoratus* strain were susceptible to ciprofloxacin. Of all quinolones tested, moxifloxacin showed the lowest MIC values in this antibiotic class. In addition, only 1 *M. odoratimimus* strain and 1* M. odoratus* were susceptible to tigecycline.

**Table 1:** MIC distribution of 43* M. odoratimimus* strains

	

*Note*: This table summarizes the resistance profiles determined for 43 *M. odoratimimus* strains. The MIC results are given in µg/ml. The number of isolates tested for each antibiotic is summarized in this table. Susceptible isolates are highlighted in green color, intermediate in yellow, and resistant isolates in red. Blue color is used to illustrate the cases with insufficient evidence (I.E.) that the antibiotic can successfully be administered to the patient. In these instances breakpoints are not provided by the EUCAST. MIC results which range below or above the scale of the E-Test are marked with “-”. Additionally, the percentages of susceptible, intermediate, and resistant strains are given.

**Table 2:** MIC distribution of 16 *M. odoratus* strains

	

*Note*: This table summarizes the resistance profiles determined for 16 *M. odoratus* strains. The MIC results are given in µg/ml. The number of isolates tested for each antibiotic is summarized in this table. Susceptible isolates are highlighted in green color, intermediate in yellow, and resistant isolates in red. Blue color is used to illustrate the cases with insufficient evidence (I.E.) that the antibiotic can successfully be administered to the patient. MIC results which range below or above the scale of the E-Test are marked with “-”. Additionally the percentages of susceptible, intermediate, and resistant strains are given. Antibiotics with insufficient evidence that they can successfully be administered to the patient are marked in analogy to the EUCAST with I.E. (insufficient evidence).

No breakpoints were available for trimethoprim/sulfamethoxazole, fosfomycin, colistin, gentamicin, amikacin, erythromycin, azithromycin, daptomycin, and rifampicin. The MIC results determined for fosfomycin, colistin, gentamicin, amikacin, and daptomycin are all at a high range. Therefore, antimicrobial resistance to these antibiotics may be assumed. Regarding trimethoprim/sulfamethoxazole, erythromycin, azithromycin, and rifampicin, the MIC results ranged from low to high levels.

### Detection of relevant β-lactamase genes

All strains were negative for genes coding for OXA-type, VIM-type, IMP-type, and NDM β-lactamases, and for KPC. The AmpC β-lactamase gene was detected in one isolate (DSM 100899, Table S2).

### Homologies of *bla*_MUS-1_ and *bla*_TUS-1_

**Fig. 1:**
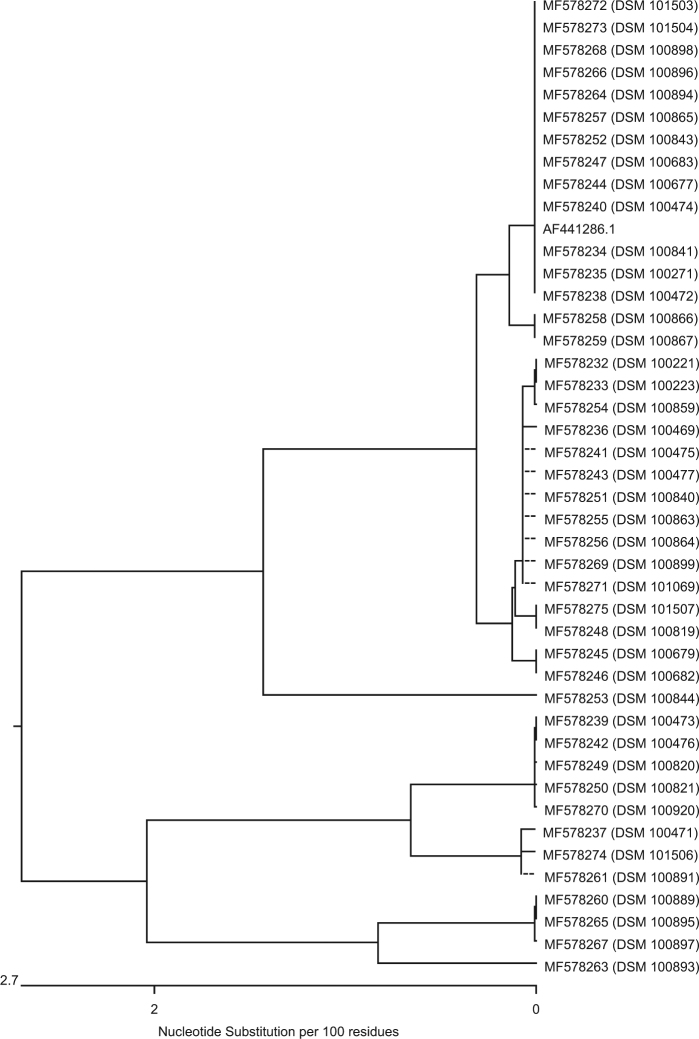
**Sequence similarity of genes derived from** ***M. odoratimimus*** **showing homology to** ***bla***_**MUS-1**_ **(AF441286.1)**

**Fig. 2:**
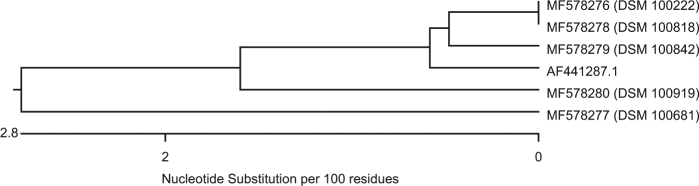
**Sequence similarity of genes derived from** ***M. odoratus*** **showing homology to** ***bla***_**TUS-1**_ **(AF441287.1)**

Genes homologous to *bla*_MUS-1_ were only detected in *M. odoratimimus*; genes homologous to *bla*_TUS-1_, only in *M. odoratus* isolates. Figure 1 is a dendrogram that presents the nucleotide sequence similarity of all genes of our *M. odoratimimus* strains that show homology to the carbapenemase MUS-1 gene of *M. odoratimimus* CIP 103073 (GenBank accession #AF441286.1). Figure 2 is also a dendrogram. It shows the nucleotide sequence similarities of our *M. odoratus* strains compared to the carbapenemase TUS-1 gene of *M. odoratus* CIP 103105 (GenBank accession #AF441287.1) in the same fashion. No DNA sequence homologies to *bla*_TUS-1_ were found in 11 out of 16 isolates (Table S3).

### Comparison of nucleotide and protein sequences

Nucleotide sequences were transferred into protein sequences using Lasergene software version 14.0.10 (DNASTAR, Madison, WI, USA). Alignments of all MUS-1 and all TUS-1 genes, as well as their protein translations, are illustrated in Figs. S1–4. When the sequences are compared, alterations are only observed at certain positions in the genes homologous to *bla*_MUS-1_ and *bla*_TUS-1_. The active centers of both enzymes, however, were not involved. This observation is confirmed by the translated protein sequences. The maximum divergence between the sequences of genes homologous to *bla*_MUS-1_ was 5.6% in the nucleotide composition and 6.5% in the amino acid (AA) composition. Additionally, the maximum divergence of genes homologous to *bla*_TUS-1_ was 5.1% at the nucleotide level and 2.8% at the AA level.

## Discussion

Bacteria of the genus *Myroides* are rare human pathogens that cause severe infections (including blood stream infections, necrotizing fasciitis, ventriculitis, and pneumonia) and affect mainly immunocompromised patients^6,7,9,18,19^. Most clinical case reports focus on *M. odoratimimus* and *M. odoratus*. Until now, whether other species of the genus *Myroides*, such as *M. pelagicus*, *M. profundi*, *M. marinus*, *M. phaeus*, *M. guanonis*, *M. xuanwuensis*, *M. injenensis*, and *M. indicus*, are pathogenic for humans has been uncertain^20–27^. *M. odoratimimus* and *M. odoratus* are described to be resistant to a large number of antibiotics. The available antimicrobial susceptibility data on *Myroides* sp. are mostly based on case reports and four systematic studies^16,28,29^. Two of these studies included only a few isolates^28,29^ and therefore are limited. Holmes et al. tested 28* F. odoratum* isolates against 15 antibiotics^16^, whereas Hu et al. summarized and compared data published in the literature and added data that previously were available only in China^17^. However, those authors did not distinguish between *M. odoratimimus* and *M. odoratus*. As far as we know, this is the only systematic study that provides reliable susceptibility data. Because *F. odoratum* was reclassified and divided into the two new species—*M. odoratimimus* and *M. odoratus*—approximately 20 years after Holmes and coworkers published their data^3,16^, resistance analysis of these two *Myroides* species must be repeated. Here, we provide susceptibility data for both *M. odoratus* and *M. odoratimimus* for the first time. Although all strains included in the study by Holmes et al. were resistant to ampicillin^16^, we found that 2.3% (*n* = 1) of the *M. odoratimimus* and 31.3% (*n* = 5) of the *M. odoratus* strains were susceptible to ampicillin (Tables 1 and 2). Additionally, 4.7% (*n* = 2) of our *M. odoratimimus* strains but none of the *M. odoratus* strains (Tables 1 and 2) were susceptible to piperacillin/tazobactam. *Myroides* sp. can show any level of susceptibility, including resistance to piperacillin/tazobactam^6,7,9,11^. However, none of our strains were susceptible to ceftazidime and cefepime (Tables 1 and 2). Our finding is also in accordance with previous reports^6,11,15^. Only Crum-Cianflone et al. reported on an *M. odoratus* isolate with intermediate susceptibility to cefepime^9^. Our data and data from previous studies show that *Myroides* sp. are resistant to aztreonam^6,7,9,11^ (Tables 1 and 2). We found that 83.7% (*n* = 36) of our *M. odoratimimus* strains and all *M. odoratus* strains were resistant to imipenem, which is consistent with data from other studies showing susceptibility^6^, intermediate resistance^9^, or resistance^7,15^. We observed that 74.4% (*n* = 32) of our *M. odoratimimus* strains and 50.0% (*n* = 8) of the *M. odoratus* strains are susceptible to meropenem (Tables 1 and 2), which has also been reported by others^6,9^. Therefore, meropenem may be more useful for the treatment of *Myroides* infections than imipenem. While investigating quinolones, we found a gradual change in susceptibility. Most strains were resistant to ciprofloxacin (Tables 1 and 2): 83.7% (*n* = 36) of the *M. odoratimimus* strains and 68.7% (*n* = 11) of the *M. odoratus* strains were resistant, whereas only 2.3% (*n* = 1) of the *M. odoratimimus* strains and 6.3% (*n* = 1) of the *M. odoratus* strains were susceptible. However, resistant, as well as susceptible, isolates have been reported before^6,7,9,18^. A larger number of isolates were susceptible to levofloxacin compared to ciprofloxacin (Tables 1 and 2), namely, 11.6% (*n* = 5) of the *M. odoratimimus* and 43.8% (*n* = 7) of the *M. odoratus* strains. Interestingly, moxifloxacin testing revealed significantly lower MIC values compared to levofloxacin and ciprofloxacin (Tables 1 and 2). In fact, only one *M. odoratus* isolate was resistant, but 90.7% (*n* = 39) of our *M. odoratimimus* isolates and 93.8% (*n* = 15) of our *M. odoratus* isolates were susceptible (Table 2). Based on these in vitro data, moxifloxacin may therefore be the best quinolone to be used. Ali et al. recently reported the successful use of moxifloxacin in a patient with canaliculitis^30^. To our knowledge, susceptibility testing for tigecycline on *Myroides* sp. has not been published before, and our data on tigecycline show that most strains of both *M.* *odoratimimus* and *M. odoratus* are resistant (Tables 1 and 2). No EUCAST guidelines exist for trimethoprim/sulfamethoxazole, fosfomycin, colistin, amikacin, erythromycin, azithromycin, daptomycin, and rifampicin. Nevertheless, we could detect very high MIC values for colistin, fosfomycin, and daptomycin for all our isolates. For this reason, *M. odoratimimus* and *M. odoratus* may be assumed to be naturally resistant to these antibiotics. Judging from the high MIC values, our strains seem to be naturally resistant against the aminoglycosides gentamicin and amikacin. Our findings are in accordance with the data reported from several laboratories working on *Myroides*^7,9,11,15,16^. The MIC values for trimethoprim/sulfamethoxazole span a wide range (for *M. odoratimimus* 0.125–8 µg/ml and for *M. odoratus* 0.5–4 µg/ml). Using different breakpoints for their antimicrobial resistance testing, Holmes et al. found both susceptible and resistant strains to trimethoprim/sulfamethoxazole^16^. Similarly, a wide range of MIC values was found for the macrolide antibiotics erythromycin and azithromycin and for rifampicin.

Very little is known about the resistance mechanisms in *Myroides* today. In 2002, Mammeri et al. identified two ß-lactamases in *Myroides* sp.—MUS-1 and TUS-1—and these metalloenzymes share 73% AA identity^31^. Based on sequence analyses, both MUS-1 and TUS-1 could be identified as members of the B1 subclass family according to the classification by Ambler^32^, which has been expanded by Galleni et al^33^. According to the kinetic studies performed by Mammeri et al., MUS-1 and TUS-1 hydrolyze all ß-lactam antibiotics except aztreonam^31^. This ability is characteristic of enzymes belonging to the subclass 3a of metallo-ß-lactamases according to the revised functional classification proposed by Bush and Jacoby in 2010^34^. In contrast, our strains were mostly resistant to aztreonam, and many were susceptible to meropenem (Tables 1 and 2). However, meropenem is characteristically hydrolyzed by group 3a ß-lactamases^34^. For this reason (resistance against aztreonam and susceptibility to meropenem), we assume that neither MUS-1 nor TUS-1 is expressed at high quantities. Therefore, additional, yet unresolved mechanisms, such as porin-mutations or other ß-lactamase hydrolyzing enzymes, likely are present and explain the extended resistance of both *M. odoratimimus* and *M. odoratus* against ß-lactam antibiotics. This observation is also supported by the fact that 11 of the *M. odoratus* strains do not contain the TUS-1 genes. However, all our *M. odoratimimus* possess MUS-1. Nevertheless, all strains of *M. odoratus*, irrespective of the presence or absence of the *bla*_TUS-1_ gene, show similar resistance profiles (Table S1). A *Myroides* strain, assigned to *M. odoratimimus* based on 16S rDNA sequencing, without *bla*_MUS-1_ has previously been reported by Dharne et al^35^. Yet this isolate was reported to be highly resistant to β-lactam antibiotics^35^. Based on molecular and phenotypic analyses, Al-Bayssari et al. proposed a new variant of a MUS ß-lactamase in 2015, which was named MUS-2 and possessed 98.78% AA homology to MUS-1^36^. However, in contrast to the work by Mammeri et al.^31,36^, these authors did not perform an in-depth analysis of the enzyme kinetics.

A comparison of the genomic data from all 48 *Myroides* strains carrying *bla*_MUS-1_/*bla*_TUS-1_ to the sequences published by Mammeri et al.^31,36^ show that the encoding genes have a fairly high homology, ranging from 94.7 to 100% for *bla*_MUS-1_ and from 94.9 to 99.3% for *bla*_TUS-1_ (Table S4 and Figs. S1, S3). At the protein level, MUS-1 shares 93.5–100% homology and TUS-1 97.2–99.6% (Table S4 and Figs. S2, S4). Notably, all 43* M. odoratimimus* strains harbor a MUS-1 encoding gene, whereas, of the 16* M. odoratus* isolates, 11 were negative for *bla*_TUS-1_. Furthermore, different clusters of “MUS” or “TUS” genes can be defined (Figs. 1, 2). Only 30.2% (*n* = 13) of our *M. odoratimimus* strains harbor genes that are genetically identical to *bla*_MUS-1_. We could not detect a gene that was 100% identical to the originally described nucleotide sequence for *bla*_TUS-1_. In other words, the variability of *M. odoratus* with respect to the gene *bla*_TUS-1_ is large. Only five genes were homologous to the sequence initially described by Mammeri et al.^31,36^, with variation ranging from 94.9 to 99.3%. Comparison of the nucleotide and AA sequences, however, suggests that genomic differences may not have an effect on the active center of the enzymes, and therefore may not affect the hydrolytic activity. Additional data from a similar comparative study on enzyme kinetics, however, would elucidate whether these sequences encode for new variants. Therefore, the results and the conclusions drawn by Al-Bayssari et al.^36^ regarding a newly described MUS-2 have to be corroborated. Notably, our *M. odoratimimus* isolate DSM 100899 harbors the gene encoding the ß-lactamase AmpC. It can be located chromosomally or on specific resistance (R-) plasmids^37^. We believe this is the first report of an AmpC encoding gene detected in *Myroides* sp. Furthermore, the R-plasmids seem to play an important role in antimicrobial resistance mechanisms in *Myroides* sp. Kono et al., for instance, reported on resistance genes against ampicillin, carbenicillin, and erythromycin which were located on an R-plasmid^38^. In addition, Kuai et al. reported on a KPC-2-positive *F. odoratum* strain in 2011^39^. In general, a variety of genes for antimicrobial resistance seems to be characteristic for *Myroides* sp. Elucidating the exact mechanisms of antimicrobial resistance, however, is a matter of ongoing and future research.

## Materials and methods

### Bacterial isolates

A collection of clinical isolates of *Myroides* strains was created over the course of 4 years (Table S5). The strains were collected during the routine diagnostics at the Institute for Medical Microbiology and Hygiene, TU Dresden, Germany. Additional strains were provided by Bodo R. Eing and Sebastian Bertram (Synlab Medizinisches Versorgungszentrum Augsburg, Augsburg, Germany). A few isolates were of veterinary origin (Table S5). The bacteria were confirmed as *M. odoratimimus* or *M. odoratus* by two independent methods: 16S rRNA gene sequencing and MALDI-TOF MS (Bruker Daltonik, Bremen, Germany). We have addressed the suitability of this identification strategy, as well as the performance and reliability of both methods, in previous studies^40,41^. In the present investigation, we included 59 strains, with 43 being identified as *M. odoratimimus* and 16 as *M. odoratus*. All strains were also deposited in the “Open Collection” of the Leibniz Institute DSMZ-German Collection of Microorganisms and Cell Cultures (Braunschweig, Germany).

### Antimicrobial susceptibility testing

The MIC tests were performed, and the results were evaluated by applying the guidelines for PK/PD (non-species related) breakpoints according to the criteria published by EUCAST (the European Committee on Antimicrobial Susceptibility Testing). EUCAST breakpoint tables were created for the interpretation of the MICs and zone diameters using Version 7.1, 2017 (http://www.eucast.org/clinical_breakpoints/). In brief, a McFarland standard of 0.5 was created for each bacterium using NaCl and a DensiCHEK densitometer (bioMérieux, Nürtingen, Germany). The suspended bacteria were plated with the help of a cotton swab on Müller-Hinton Agar (Oxoid Deutschland, Wesel, Germany). Then, E-Test strips (bestbion, Cologne, Germany) for each antibiotic were placed on the agar plates. The plates were incubated for 18 ± 2 h at 37 °C and 5% CO_2_. Then, the MIC results were determined. The ATCC strains *Escherichia coli* ATCC^®^ 25922™, *Pseudomonas aeruginosa* ATCC^®^ 27853™, and *Staphylococcus aureus* ATCC^®^ 25923™ served as the controls. All E-Test strips used in this study and their MIC ranges are listed in Table S6.

### Real-time PCR analysis for β-lactamase gene detection

All samples were cultivated on blood agar plates (25 °C, 24 h), and DNA was extracted with a QB-EX-50 QuickBlue extraction kit using magnetic nanoparticles (Q-Bioanalytic, Bremerhaven, Germany). Real-time PCR analysis was carried out on all isolates to evaluate the prevalence of the *bla*_OXA_, *bla*_VIM_, *bla*_IMP_,* bla*_AmpC_, and *bla*_KPC_ carbapenemase genes and the *bla*_NDM_ metallo-ß-lactamase gene. QuickBlue RealQuick PCR master mix—SYBR Green (QB-RT-ESBL-50-SYBR, Q-Bioanalytic) for carbapenemase gene detection was prepared in 15 µl of master mix that included Taq polymerase, buffer, dNTPs, the specific primer set, and SYBR Green. The amplification and detection of the metallo-ß-lactamase *bla*_NDM_ amplicon was carried out using QuickBlue RealQuick PCR master mix (QB-RT-ESBL-50, Q-Bioanalytic), which included Taq polymerase, buffer, dNTPs, specific primer set, and probe (Fam-labeled). Two microliters of DNA template (5–10 ng genomic DNA) was added to each PCR application, bringing the total volume to 17 µl. Amplification reactions were carried out using a real-time thermal cycler (LightCycler 480 II, Roche Deutschland, Grenzach-Wyhlen, Germany) with the following program: one cycle of 5 min at 95 °C, 35 cycles with denaturation at 95 °C for 15 s and annealing at 60 °C for 20 s; and an extension at 72 °C for 15 s. Data were analyzed using the Roche LightCycler Software 1.5. Primer sets were designed with Integrated DNA Technologies “PrimerQuest Tool” (https://eu.idtdna.com/PrimerQuest/Home/Index) using genomic sequences from NCBI GenBank. Multiple alignments from several ß-lactamase genes were analyzed with the Clustal Omega program (https://www.ebi.ac.uk/Tools/msa/clustalo/), and the primer sets were optimized to ensure detection of a broad range of bacterial resistance genes. *Klebsiella pneumoniae* NCTC 13443 (*bla*_NDM_), *K. pneumoniae* NCTC 13440 (*bla*_VIM-1_), *K. pneumoniae* NCTC 13442 (*bla*_OXA-48_), *K. pneumoniae* BAA 1705 (*bla*_KPC_), *E. coli* NCTC 13476 (*bla*_IMP_), and *Enterobacter cloacae* BAA 1143 (*bla*_AmpC_) strains were used as negative and positive controls. The results are illustrated in Table S2.

### Detection of MUS-1 and TUS-1 encoding genes using a whole-genome sequencing approach

Libraries for whole-genome sequencing were prepared with Nextera XT DNA Library Prep Kit (Illumina, San Diego, CA, USA). Sequencing was performed on the Illumina HiSeq 2500 (Illumina) for 100 cycles in both directions at the Helmholtz Centre for Infection Research (Braunschweig, Germany). Hereby, coverages of approximately 100× were obtained for each of the isolates. Assembly of short read genome data was performed using Velvet 1.2.10^42^ with a kmer size of 61 for all isolates. Resulting contig sets were screened for known antibiotic resistance genes querying the ResFinder 2.1 database^43^ in an automated approach. Hereby, minimum values of 60% query coverage and 90% identity have been used as search parameters. A comparative analysis of the DNA sequences obtained was performed using the Molecular Evolutionary Genetics Analysis software package version 7^44^. Protein sequences were created using DNASTAR Lasergene 14.0.10. (DNASTAR). Average nucleotide identity (ANI) values have been calculated on the whole-genome level using the software FastANI (https://github.com/ParBLiSS/FastANI) (Table S3). Therefore, all *M. odoratimimus* strains used in this study were compared with *M. odoratimimus* CCUG39352^T^ (draft assembly ASM148541v1, https://www.ncbi.nlm.nih.gov/assembly/GCF_001485415.1), and all *M. odoratus* strains were compared with *M. odoratus* DSM 2801^T^ (GenBank accession #CM001437).

## Electronic supplementary material

Table S1 (DOCX 110 kb)

Table S2 (DOCX 73 kb)

Table S3 (DOCX 82 kb)

Table S4 (DOCX 68 kb)

Table S5(DOCX 27 kb)

Table S6 (DOCX 34 kb)

Figure S1(PNG 767 kb)

Figure S2(PNG 479 kb)

Figure S3(PNG 118 kb)

Figure S4(PNG 74 kb)
