# Supplementary material for: Whole-genome sequencing of a large collection of Myroides odoratimimus and Myroides odoratus isolates and antimicrobial susceptibility studies
Source: Emerg Microbes Infect. 2018 Apr 4;7:61. doi: 10.1038/s41426-018-0061-x (PMC5884818; doi:10.1038/s41426-018-0061-x)
Supplement: Supplementary file 1 — Table S1 (DOCX 110 kb) [file 41426_2018_61_MOESM1_ESM.docx]

Table S1: MIC values of 59 *Myroides* sp. strains tested.

| **DSM number** | **Species** | **Ampicillin** | **Piperacillin/ tzobactam** | **Ceftazidim** | **Cefepim** | **Aztreonam** | **Imipenem** | **Meropenem** | **Ciprofloxacin** | **Levofloxacin** | **Moxifloxacin** | **Trimethoprim/ sulfamethoxazole** | **Tigecycline** | **Fosfomycin** | **Colistin** | **Gentamcin** | **Amikacin** | **Erythromycin** | **Acithromycin** | **Daptomycin** | **Rifampicin** |
| --- | --- | --- | --- | --- | --- | --- | --- | --- | --- | --- | --- | --- | --- | --- | --- | --- | --- | --- | --- | --- | --- |
| 100221 | *M. odoratimimus* | 64 | 32 | 256 | 32 | 256 | 32 | 2 | 32 | 32 | 1 | 2 | 2 | 1024 | 256 | 1024 | 256 | 16 | 0.5 | 256 | 1 |
| 100223 | *M. odoratimimus* | 4 | 32 | 32 | 32 | 8 | 32 | 1 | 0.5 | 1 | 0.032 | 2 | 1 | 1024 | 256 | 64 | 128 | 0.5 | 0.25 | 256 | 2 |
| 100271 | *M. odoratimimus* | 16 | 128 | 64 | 32 | 64 | 32 | 4 | 1 | 1 | 0.032 | 2 | 1 | 1024 | 256 | 1024 | 256 | 2 | 1 | 256 | 4 |
| 100469 | *M. odoratimimus* | 16 | 32 | 128 | 16 | 256 | 32 | 2 | 1 | 2 | 0.064 | 4 | 2 | 1024 | 256 | 64 | 256 | 4 | 2 | 256 | 4 |
| 100471 | *M. odoratimimus* | 16 | 128 | 64 | 32 | 64 | 32 | 4 | 1 | 1 | 0.032 | 2 | 1 | 1024 | 256 | 1024 | 256 | 2 | 1 | 256 | 4 |
| 100472 | *M. odoratimimus* | 8 | 64 | 256 | 16 | 16 | 32 | 2 | 1 | 1 | 0.032 | 2 | 8 | 1024 | 256 | 256 | 256 | 1 | 1 | 256 | 4 |
| 100473 | *M. odoratimimus* | 8 | 64 | 128 | 16 | 8 | 32 | 2 | 1 | 2 | 0.064 | 4 | 2 | 1024 | 256 | 1024 | 256 | 4 | 2 | 256 | 1 |
| 100474 | *M. odoratimimus* | 8 | 64 | 128 | 8 | 8 | 32 | 32 | 2 | 1 | 0.064 | 4 | 2 | 1024 | 256 | 64 | 256 | 4 | 2 | 256 | 1 |
| 100475 | *M. odoratimimus* | 32 | 256 | 256 | 64 | 32 | 32 | 4 | 4 | 4 | 0.064 | 2 | 16 | 1024 | 256 | 1024 | 256 | 4 | 1 | 256 | 1 |
| 100476 | *M. odoratimimus* | 16 | 64 | 64 | 16 | 16 | 32 | 2 | 1 | 4 | 0.064 | 2 | 2 | 1024 | 256 | 64 | 128 | 4 | 1 | 256 | 2 |
| 100477 | *M. odoratimimus* | 16 | 128 | 256 | 32 | 64 | 32 | 8 | 1 | 2 | 0.064 | 4 | 1 | 1024 | 256 | 1024 | 256 | 2 | 1 | 256 | 2 |
| 100677 | *M. odoratimimus* | 8 | 16 | 32 | 256 | 32 | 2 | 2 | 0.5 | 1 | 0.064 | 2 | 2 | 1024 | 256 | 1024 | 256 | 1 | 0.125 | 256 | 1 |
| 100679 | *M. odoratimimus* | 64 | 64 | 256 | 32 | 256 | 4 | 2 | 2 | 1 | 0.25 | 2 | 1 | 1024 | 256 | 1024 | 256 | 8 | 8 | 256 | 0.5 |
| 100682 | *M. odoratimimus* | 8 | 32 | 256 | 32 | 256 | 4 | 2 | 2 | 2 | 0.125 | 2 | 1 | 1024 | 256 | 1024 | 256 | 4 | 256 | 256 | 0.5 |
| 100683 | *M. odoratimimus* | 16 | 128 | 256 | 32 | 256 | 32 | 8 | 8 | 4 | 0.5 | 2 | 0.5 | 1024 | 256 | 1024 | 256 | 8 | 4 | 256 | 2 |
| 100819 | *M. odoratimimus* | 8 | 32 | 128 | 16 | 64 | 32 | 2 | 1 | 1 | 0.064 | 8 | 2 | 1024 | 256 | 1024 | 256 | 2 | 0.5 | 256 | 2 |
| 100820 | *M. odoratimimus* | 8 | 8 | 32 | 256 | 256 | 32 | 2 | 1 | 1 | 0.125 | 4 | 1 | 1024 | 256 | 1024 | 256 | 2 | 1 | 256 | 1 |
| 100821 | *M. odoratimimus* | 4 | 1 | 16 | 4 | 4 | 32 | 1 | 1 | 1 | 0.25 | 0.5 | 0.25 | 1024 | 256 | 1024 | 256 | 2 | 2 | 256 | 0.5 |
| 100840 | *M. odoratimimus* | 4 | 16 | 8 | 256 | 8 | 2 | 1 | 0.5 | 1 | 0.064 | 1 | 8 | 1024 | 256 | 1024 | 256 | 32 | 8 | 256 | 1 |
| 100841 | *M. odoratimimus* | 8 | 16 | 256 | 16 | 8 | 8 | 1 | 2 | 2 | 0.25 | 1 | 1 | 1024 | 256 | 1024 | 256 | 8 | 2 | 256 | 2 |
| 100843 | *M. odoratimimus* | 16 | 16 | 256 | 16 | 256 | 2 | 2 | 1 | 1 | 0.064 | 4 | 0.5 | 1024 | 256 | 1024 | 256 | 2 | 0.5 | 256 | 2 |
| 100844 | *M. odoratimimus* | 4 | 4 | 32 | 256 | 256 | 2 | 1 | 0.5 | 0.5 | 0.5 | 4 | 1 | 1024 | 256 | 1024 | 256 | 2 | 0.125 | 256 | 1 |
| 100859 | *M. odoratimimus* | 64 | 16 | 64 | 16 | 256 | 32 | 0.25 | 0.125 | 0.5 | 0.5 | 0.125 | 2 | 1024 | 64 | 1024 | 256 | 256 | 16 | 256 | 1 |
| 100863 | *M. odoratimimus* | 128 | 64 | 256 | 64 | 32 | 32 | 2 | 1 | 1 | 0.032 | 2 | 1 | 1024 | 256 | 256 | 256 | 1 | 0.5 | 256 | 1 |
| 100864 | *M. odoratimimus* | 32 | 32 | 256 | 256 | 256 | 32 | 2 | 32 | 32 | 0.064 | 2 | 2 | 1024 | 256 | 1024 | 256 | 16 | 0.5 | 256 | 1 |
| 100865 | *M. odoratimimus* | 8 | 32 | 128 | 8 | 16 | 32 | 1 | 1 | 1 | 0.032 | 2 | 2 | 1024 | 256 | 512 | 256 | 2 | 1 | 256 | 1 |
| 100866 | *M. odoratimimus* | 4 | 64 | 128 | 8 | 64 | 32 | 1 | 1 | 1 | 0.016 | 2 | 2 | 1024 | 256 | 1024 | 256 | 0.5 | 0.5 | 256 | 1 |
| 100867 | *M. odoratimimus* | 8 | 64 | 64 | 16 | 64 | 32 | 1 | 1 | 1 | 0.032 | 2 | 2 | 1024 | 256 | 1024 | 256 | 1 | 2 | 256 | 1 |
| 100889 | *M. odoratimimus* | 16 | 16 | 128 | 16 | 32 | 32 | 2 | 1 | 0.5 | 0.064 | 2 | 4 | 1024 | 256 | 256 | 256 | 4 | 1 | 256 | 4 |
| 100891 | *M. odoratimimus* | 8 | 32 | 128 | 16 | 64 | 32 | 2 | 1 | 1 | 0.064 | 8 | 2 | 1024 | 256 | 1024 | 256 | 2 | 0.5 | 256 | 2 |
| 100893 | *M. odoratimimus* | 256 | 64 | 64 | 64 | 32 | 32 | 2 | 1 | 1 | 0.032 | 4 | 2 | 1024 | 256 | 1024 | 256 | 128 | 16 | 256 | 8 |
| 100894 | *M. odoratimimus* | 16 | 32 | 64 | 32 | 32 | 32 | 2 | 1 | 1 | 0.032 | 4 | 1 | 1024 | 256 | 128 | 256 | 1 | 1 | 256 | 2 |
| 100895 | *M. odoratimimus* | 4 | 32 | 128 | 32 | 16 | 32 | 4 | 0.5 | 1 | 0.032 | 4 | 2 | 1024 | 256 | 1024 | 256 | 1 | 1 | 256 | 1 |
| 100896 | *M. odoratimimus* | 8 | 64 | 64 | 16 | 16 | 32 | 4 | 0.5 | 0.5 | 0.016 | 2 | 1 | 1024 | 256 | 1024 | 256 | 1 | 1 | 256 | 2 |
| 100897 | *M. odoratimimus* | 16 | 32 | 128 | 16 | 16 | 32 | 8 | 32 | 32 | 0.032 | 2 | 1 | 1024 | 256 | 1024 | 256 | 256 | 256 | 256 | 8 |
| 100898 | *M. odoratimimus* | 16 | 32 | 128 | 8 | 64 | 32 | 2 | 1 | 1 | 0.016 | 2 | 2 | 1024 | 256 | 1024 | 256 | 1 | 2 | 256 | 2 |
| 100899 | *M. odoratimimus* | 16 | 16 | 128 | 16 | 32 | 32 | 2 | 1 | 0.5 | 0.064 | 2 | 4 | 1024 | 256 | 256 | 256 | 4 | 1 | 256 | 4 |
| 100920 | *M. odoratimimus* | 16 | 16 | 32 | 16 | 16 | 32 | 2 | 1 | 2 | 0.25 | 8 | 1 | 1024 | 256 | 32 | 128 | 4 | 2 | 256 | 2 |
| 101069 | *M. odoratimimus* | 2 | 32 | 256 | 16 | 32 | 32 | 0.25 | 2 | 2 | 0.125 | 0.5 | 1 | 1024 | 256 | 1024 | 256 | 2 | 8 | 256 | 1 |
| 101503 | *M. odoratimimus* | 8 | 128 | 128 | 128 | 8 | 32 | 1 | 1 | 1 | 0.064 | 8 | 8 | 1024 | 256 | 1024 | 256 | 1 | 1 | 256 | 2 |
| 101504 | *M. odoratimimus* | 16 | 32 | 256 | 16 | 64 | 32 | 2 | 1 | 2 | 0.064 | 4 | 1 | 1024 | 256 | 1024 | 256 | 4 | 16 | 256 | 1 |
| 101506 | *M. odoratimimus* | 16 | 32 | 256 | 16 | 32 | 32 | 4 | 2 | 2 | 0.25 | 2 | 0.5 | 1024 | 256 | 1024 | 256 | 2 | 8 | 256 | 2 |
| 101507 | *M. odoratimimus* | 16 | 32 | 256 | 16 | 32 | 32 | 4 | 2 | 2 | 0.25 | 1 | 0.5 | 1024 | 256 | 1024 | 256 | 2 | 16 | 256 | 1 |
| 100222 | *M. odoratus* | 16 | 64 | 64 | 128 | 256 | 32 | 1 | 1 | 1 | 0.064 | 0.5 | 2 | 1024 | 256 | 1024 | 256 | 1 | 1 | 256 | 32 |
| 100470 | *M. odoratus* | 2 | 64 | 128 | 16 | 256 | 32 | 0.25 | 1 | 0.5 | 0.016 | 4 | 1 | 1024 | 256 | 128 | 128 | 2 | 1 | 256 | 1 |
| 100678 | *M. odoratus* | 256 | 256 | 64 | 32 | 256 | 32 | 8 | 2 | 1 | 0.25 | 2 | 1 | 1024 | 256 | 1024 | 256 | 8 | 8 | 256 | 1 |
| 100680 | *M. odoratus* | 128 | 256 | 256 | 32 | 256 | 32 | 32 | 2 | 1 | 0.125 | 2 | 0.5 | 1024 | 256 | 1024 | 256 | 2 | 4 | 256 | 1 |
| 100681 | *M. odoratus* | 128 | 256 | 256 | 32 | 256 | 32 | 16 | 2 | 1 | 0.125 | 2 | 0.25 | 1024 | 256 | 1024 | 256 | 4 | 4 | 256 | 2 |
| 100817 | *M. odoratus* | 64 | 256 | 256 | 32 | 256 | 32 | 32 | 1 | 0.5 | 0.125 | 1 | 0.5 | 1024 | 256 | 1024 | 256 | 0.5 | 1 | 256 | 32 |
| 100818 | *M. odoratus* | 8 | 64 | 128 | 32 | 256 | 32 | 16 | 32 | 32 | 4 | 2 | 1 | 1024 | 256 | 1024 | 256 | 2 | 16 | 256 | 8 |
| 100839 | *M. odoratus* | 128 | 256 | 32 | 256 | 256 | 32 | 32 | 0.25 | 0.5 | 0.064 | 0.5 | 0.5 | 1024 | 256 | 1024 | 256 | 1 | 0.5 | 256 | 16 |
| 100842 | *M. odoratus* | 16 | 32 | 256 | 64 | 256 | 8 | 2 | 1 | 1 | 0.125 | 0.25 | 0.5 | 1024 | 256 | 1024 | 256 | 1 | 0.125 | 256 | 4 |
| 100857 | *M. odoratus* | 16 | 256 | 256 | 16 | 32 | 32 | 8 | 1 | 1 | 0.016 | 1 | 8 | 1024 | 256 | 1024 | 256 | 1 | 1 | 256 | 16 |
| 100858 | *M. odoratus* | 2 | 64 | 64 | 16 | 64 | 8 | 0.25 | 1 | 1 | 0.016 | 4 | 4 | 1024 | 256 | 128 | 64 | 2 | 0.5 | 256 | 4 |
| 100860 | *M. odoratus* | 0.5 | 64 | 128 | 16 | 256 | 16 | 0.25 | 0.5 | 0.5 | 0.016 | 2 | 1 | 1024 | 32 | 32 | 256 | 1 | 1 | 256 | 0.25 |
| 100861 | *M. odoratus* | 0.5 | 128 | 128 | 16 | 256 | 16 | 0.5 | 0.5 | 0.5 | 0.032 | 2 | 0.5 | 1024 | 64 | 128 | 256 | 2 | 0.5 | 256 | 0.5 |
| 100862 | *M. odoratus* | 16 | 32 | 256 | 32 | 64 | 32 | 4 | 2 | 2 | 0.25 | 2 | 1 | 1024 | 256 | 1024 | 256 | 1 | 16 | 256 | 1 |
| 100890 | *M. odoratus* | 2 | 64 | 64 | 16 | 64 | 4 | 0.25 | 0.5 | 0.5 | 0.016 | 2 | 1 | 1024 | 128 | 1024 | 256 | 2 | 0.5 | 256 | 1 |
| 100919 | *M. odoratus* | 8 | 128 | 64 | 128 | 32 | 32 | 1 | 0.5 | 0.5 | 0.032 | 1 | 1 | 1024 | 256 | 1024 | 256 | 1 | 0.25 | 256 | 8 |
