# Supplementary material for: Whole-genome sequencing of a large collection of Myroides odoratimimus and Myroides odoratus isolates and antimicrobial susceptibility studies
Source: Emerg Microbes Infect. 2018 Apr 4;7:61. doi: 10.1038/s41426-018-0061-x (PMC5884818; doi:10.1038/s41426-018-0061-x)
Supplement: Supplementary file 2 — Table S2 (DOCX 73 kb) [file 41426_2018_61_MOESM2_ESM.docx]

Table S2: Distribution of genes encoding for important carbapene­mases in *Myroides* sp.

| DSM number | Species | OXA | VIM | IMP | AmpC | KPC | NDM |
| --- | --- | --- | --- | --- | --- | --- | --- |
| 100221 | *M. odoratimimus* | Ø | Ø | Ø | Ø | Ø | Ø |
| 100223 | *M. odoratimimus* | Ø | Ø | Ø | Ø | Ø | Ø |
| 100271 | *M. odoratimimus* | Ø | Ø | Ø | Ø | Ø | Ø |
| 100469 | *M. odoratimimus* | Ø | Ø | Ø | Ø | Ø | Ø |
| 100471 | *M. odoratimimus* | Ø | Ø | Ø | Ø | Ø | Ø |
| 100472 | *M. odoratimimus* | Ø | Ø | Ø | Ø | Ø | Ø |
| 100473 | *M. odoratimimus* | Ø | Ø | Ø | Ø | Ø | Ø |
| 100474 | *M. odoratimimus* | Ø | Ø | Ø | Ø | Ø | Ø |
| 100475 | *M. odoratimimus* | Ø | Ø | Ø | Ø | Ø | Ø |
| 100476 | *M. odoratimimus* | Ø | Ø | Ø | Ø | Ø | Ø |
| 100477 | *M. odoratimimus* | Ø | Ø | Ø | Ø | Ø | Ø |
| 100677 | *M. odoratimimus* | Ø | Ø | Ø | Ø | Ø | Ø |
| 100679 | *M. odoratimimus* | Ø | Ø | Ø | Ø | Ø | Ø |
| 100682 | *M. odoratimimus* | Ø | Ø | Ø | Ø | Ø | Ø |
| 100683* | *M. odoratimimus* | Ø | Ø | Ø | Ø | Ø | Ø |
| 100819 | *M. odoratimimus* | Ø | Ø | Ø | Ø | Ø | Ø |
| 100820 | *M. odoratimimus* | Ø | Ø | Ø | Ø | Ø | Ø |
| 100821 | *M. odoratimimus* | Ø | Ø | Ø | Ø | Ø | Ø |
| 100840 | *M. odoratimimus* | Ø | Ø | Ø | Ø | Ø | Ø |
| 100841 | *M. odoratimimus* | Ø | Ø | Ø | Ø | Ø | Ø |
| 100843 | *M. odoratimimus* | Ø | Ø | Ø | Ø | Ø | Ø |
| 100844 | *M. odoratimimus* | Ø | Ø | Ø | Ø | Ø | Ø |
| 100859 | *M. odoratimimus* | Ø | Ø | Ø | Ø | Ø | Ø |
| 100863 | *M. odoratimimus* | Ø | Ø | Ø | Ø | Ø | Ø |
| 100864 | *M. odoratimimus* | Ø | Ø | Ø | Ø | Ø | Ø |
| 100865 | *M. odoratimimus* | Ø | Ø | Ø | Ø | Ø | Ø |
| 100866 | *M. odoratimimus* | Ø | Ø | Ø | Ø | Ø | Ø |
| 100867 | *M. odoratimimus* | Ø | Ø | Ø | Ø | Ø | Ø |
| 100889 | *M. odoratimimus* | Ø | Ø | Ø | Ø | Ø | Ø |
| 100891 | *M. odoratimimus* | Ø | Ø | Ø | Ø | Ø | Ø |
| 100893 | *M. odoratimimus* | Ø | Ø | Ø | Ø | Ø | Ø |
| 100894 | *M. odoratimimus* | Ø | Ø | Ø | Ø | Ø | Ø |
| 100895 | *M. odoratimimus* | Ø | Ø | Ø | Ø | Ø | Ø |
| 100896 | *M. odoratimimus* | Ø | Ø | Ø | Ø | Ø | Ø |
| 100897 | *M. odoratimimus* | Ø | Ø | Ø | Ø | Ø | Ø |
| 100898 | *M. odoratimimus* | Ø | Ø | Ø | Ø | Ø | Ø |
| 100899 | *M. odoratimimus* | Ø | Ø | Ø | + | Ø | Ø |
| 100920 | *M. odoratimimus* | Ø | Ø | Ø | Ø | Ø | Ø |
| 101069* | *M. odoratimimus* | Ø | Ø | Ø | Ø | Ø | Ø |
| 101503 | *M. odoratimimus* | Ø | Ø | Ø | Ø | Ø | Ø |
| 101504* | *M. odoratimimus* | Ø | Ø | Ø | Ø | Ø | Ø |
| 101506* | *M. odoratimimus* | Ø | Ø | Ø | Ø | Ø | Ø |
| 101507* | *M. odoratimimus* | Ø | Ø | Ø | Ø | Ø | Ø |
| 100222 | *M. odoratus* | Ø | Ø | Ø | Ø | Ø | Ø |
| 100470 | *M. odoratus* | Ø | Ø | Ø | Ø | Ø | Ø |
| 100678 | *M. odoratus* | Ø | Ø | Ø | Ø | Ø | Ø |
| 100680 | *M. odoratus* | Ø | Ø | Ø | Ø | Ø | Ø |
| 100681 | *M. odoratus* | Ø | Ø | Ø | Ø | Ø | Ø |
| 100817 | *M. odoratus* | Ø | Ø | Ø | Ø | Ø | Ø |
| 100818 | *M. odoratus* | Ø | Ø | Ø | Ø | Ø | Ø |
| 100839 | *M. odoratus* | Ø | Ø | Ø | Ø | Ø | Ø |
| 100842 | *M. odoratus* | Ø | Ø | Ø | Ø | Ø | Ø |
| 100857 | *M. odoratus* | Ø | Ø | Ø | Ø | Ø | Ø |
| 100858 | *M. odoratus* | Ø | Ø | Ø | Ø | Ø | Ø |
| 100860 | *M. odoratus* | Ø | Ø | Ø | Ø | Ø | Ø |
| 100861 | *M. odoratus* | Ø | Ø | Ø | Ø | Ø | Ø |
| 100862* | *M. odoratus* | Ø | Ø | Ø | Ø | Ø | Ø |
| 100890 | *M. odoratus* | Ø | Ø | Ø | Ø | Ø | Ø |
| 100919 | *M. odoratus* | Ø | Ø | Ø | Ø | Ø | Ø |
| BAA-1143 | *E. cloacae* | Ø | Ø | Ø | + | Ø | Ø |
| NCTC 13476 | *E. coli* | Ø | Ø | + | Ø | Ø | Ø |
| BAA-1705 | *K. pneumoniae* | Ø | Ø | Ø | Ø | + | Ø |
| NCTC 13440 | *K. pneumoniae* | Ø | + | Ø | Ø | Ø | Ø |
| NCTC 13442 | *K. pneumoniae* | + | Ø | Ø | Ø | Ø | Ø |
| NCTC 13443 | *K. pneumoniae* | Ø | Ø | Ø | Ø | Ø | + |

*Data obtained from whole genome sequencing
