# Supplementary material for: Whole-genome sequencing of a large collection of Myroides odoratimimus and Myroides odoratus isolates and antimicrobial susceptibility studies
Source: Emerg Microbes Infect. 2018 Apr 4;7:61. doi: 10.1038/s41426-018-0061-x (PMC5884818; doi:10.1038/s41426-018-0061-x)
Supplement: Supplementary file 3 — Table S3 (DOCX 82 kb) [file 41426_2018_61_MOESM3_ESM.docx]

Table S3: Average nucleotide identity and distribution of *bla*_MUS-1_ and *bla*_TUS-1_ homologs in the strains investigated.

| DSM number | Species | % values ANI* | *bla*_MUS-1_ | *bla*_TUS-1_ | Neither *bla*_MUS-1_ nor *bla*_TUS-1_ | % A+T content | % G+C content | GeneBank  accession # |
| --- | --- | --- | --- | --- | --- | --- | --- | --- |
| 100221 | *M. odoratimimus* | 98.3 | x |  |  | 63.83 | 36.17 | MF578232 |
| 100223 | *M. odoratimimus* | 97.9 | x |  |  | 63.83 | 36.17 | MF578233 |
| 100271 | *M. odoratimimus* | 98.9 | x |  |  | 63.83 | 36.17 | MF578235 |
| 100469 | *M. odoratimimus* | 97.6 | x |  |  | 63.70 | 36.30 | MF578236 |
| 100471 | *M. odoratimimus* | 95.2 | x |  |  | 63.83 | 36.17 | MF578237 |
| 100472 | *M. odoratimimus* | 98.9 | x |  |  | 63.83 | 36.17 | MF578238 |
| 100473 | *M. odoratimimus* | 94.9 | x |  |  | 63.70 | 36.30 | MF578239 |
| 100474 | *M. odoratimimus* | 98.9 | x |  |  | 63.83 | 36.17 | MF578240 |
| 100475 | *M. odoratimimus* | 97.9 | x |  |  | 63.97 | 36.03 | MF578241 |
| 100476 | *M. odoratimimus* | 94.9 | x |  |  | 63.70 | 36.30 | MF578242 |
| 100477 | *M. odoratimimus* | 97.9 | x |  |  | 63.97 | 36.03 | MF578243 |
| 100677 | *M. odoratimimus* | 98.6 | x |  |  | 63.83 | 36.17 | MF578244 |
| 100679 | *M. odoratimimus* | 98.6 | x |  |  | 63.83 | 36.17 | MF578245 |
| 100682 | *M. odoratimimus* | 98.6 | x |  |  | 63.83 | 36.17 | MF578246 |
| 100683 | *M. odoratimimus* | 99.2 | x |  |  | 63.83 | 36.17 | MF578247 |
| 100819 | *M. odoratimimus* | 97.2 | x |  |  | 63.83 | 36.17 | MF578248 |
| 100820 | *M. odoratimimus* | 94.9 | x |  |  | 63.70 | 36.30 | MF578249 |
| 100821 | *M. odoratimimus* | 95.0 | x |  |  | 63.70 | 36.30 | MF578250 |
| 100840 | *M. odoratimimus* | 98.0 | x |  |  | 63.97 | 36.03 | MF578251 |
| 100841 | *M. odoratimimus* | 98.8 | x |  |  | 63.83 | 36.17 | MF578234 |
| 100843 | *M. odoratimimus* | 98.9 | x |  |  | 63.83 | 36.17 | MF578252 |
| 100844 | *M. odoratimimus* | 97.9 | x |  |  | 63.43 | 36,57 | MF578253 |
| 100859 | *M. odoratimimus* | 98.2 | x |  |  | 63.83 | 36.17 | MF578254 |
| 100863 | *M. odoratimimus* | 98.0 | x |  |  | 63.97 | 36.03 | MF578255 |
| 100864 | *M. odoratimimus* | 98.1 | x |  |  | 63.97 | 36.03 | MF578256 |
| 100865 | *M. odoratimimus* | 99.1 | x |  |  | 63.83 | 36.17 | MF578257 |
| 100866 | *M. odoratimimus* | 97.9 | x |  |  | 63.56 | 36.44 | MF578258 |
| 100867 | *M. odoratimimus* | 97.8 | x |  |  | 63.56 | 36.44 | MF578259 |
| 100889 | *M. odoratimimus* | 98.3 | x |  |  | 63.97 | 36.03 | MF578260 |
| 100891 | *M. odoratimimus* | 97.9 | x |  |  | 63.83 | 36.17 | MF578261 |
| 100893 | *M. odoratimimus* | 97.4 | x |  |  | 63.70 | 36.30 | MF578263 |
| 100894 | *M. odoratimimus* | 99.0 | x |  |  | 63.83 | 36.17 | MF578264 |
| 100895 | *M. odoratimimus* | 98.5 | x |  |  | 64.25 | 35.75 | MF578265 |
| 100896 | *M. odoratimimus* | 98.7 | x |  |  | 63.83 | 36.17 | MF578266 |
| 100897 | *M. odoratimimus* | 98.8 | x |  |  | 63.97 | 36.03 | MF578267 |
| 100898 | *M. odoratimimus* | 98.9 | x |  |  | 63.83 | 36.17 | MF578268 |
| 100899 | *M. odoratimimus* | 98.3 | x |  |  | 63.97 | 36.03 | MF578269 |
| 100920 | *M. odoratimimus* | 95.0 | x |  |  | 63.70 | 36.30 | MF578270 |
| 101069 | *M. odoratimimus* | 98.1 | x |  |  | 63.97 | 36.03 | MF578271 |
| 101503 | *M. odoratimimus* | 98.9 | x |  |  | 63.83 | 36.17 | MF578272 |
| 101504 | *M. odoratimimus* | 98.7 | x |  |  | 63.83 | 36.17 | MF578273 |
| 101506 | *M. odoratimimus* | 95.0 | x |  |  | 63.97 | 36.03 | MF578274 |
| 101507 | *M. odoratimimus* | 97.2 | x |  |  | 63.83 | 36.17 | MF578275 |
| 100222 | *M. odoratus* | 97.8 |  | x |  | 61.45 | 38.55 | MF578276 |
| 100470 | *M. odoratus* | 84.2 |  |  | X |  |  |  |
| 100678 | *M. odoratus* | 83.7 |  |  | X |  |  |  |
| 100680 | *M. odoratus* | 91.4 |  |  | X |  |  |  |
| 100681 | *M. odoratus* | 95.8 |  | x |  | 61.85 | 38.15 | MF578277 |

| 100817 | *M. odoratus* | 91.4 |  |  | X |  |  |  |
| --- | --- | --- | --- | --- | --- | --- | --- | --- |
| 100818 | *M. odoratus* | 97.8 |  | x |  | 61.45 | 38.55 | MF578278 |
| 100839 | *M. odoratus* | 91.4 |  |  | X |  |  |  |
| 100842 | *M. odoratus* | 98.2 |  | x |  | 61.31 | 38.69 | MF578279 |
| 100857 | *M. odoratus* | 91.5 |  |  | X |  |  |  |
| 100858 | *M. odoratus* | 83.1 |  |  | X |  |  |  |
| 100860 | *M. odoratus* | 84.4 |  |  | X |  |  |  |
| 100861 | *M. odoratus* | 84.3 |  |  | X |  |  |  |
| 100862 | *M. odoratus* | 84.3 |  |  | X |  |  |  |
| 100890 | *M. odoratus* | 83.1 |  |  | x |  |  |  |
| 100919 | *M. odoratus* | 95.8 |  | x |  | 62.12 | 37.88 | MF578280 |

Average nucleotide identity (ANI) values have been calculated on whole genome level using the software FastANI (https://github.com/ParBLiSS/FastANI). Therefore, all *M. odoratimimus* strains comprised within this study were compared against *M. odoratimimus* CCUG39352^T^ (draft assembly ASM148541v1, https://www.ncbi.nlm.nih.gov/assembly/GCF_001485415.1) and all *M. odoratus* strains against *M. odoratus* DSM 2801^T^ (GenBank accession # CM001437).
