# Supplementary material for: Whole-genome sequencing of a large collection of Myroides odoratimimus and Myroides odoratus isolates and antimicrobial susceptibility studies
Source: Emerg Microbes Infect. 2018 Apr 4;7:61. doi: 10.1038/s41426-018-0061-x (PMC5884818; doi:10.1038/s41426-018-0061-x)
Supplement: Supplementary file 4 — Table S4 (DOCX 68 kb) [file 41426_2018_61_MOESM4_ESM.docx]

Table S4: *In silico* protein analysis of MUS-1 and TUS-1.

| DSM number | Species | % identity to *bla*_MUS-1_/ *bla*_TUS-1_ | Number of AA | % identity to MUS-1/ TUS-1 | Strongly basic AA | Strongly acidic AA | Hydro-phobic AA | Polar AA | Isoelectric Point |
| --- | --- | --- | --- | --- | --- | --- | --- | --- | --- |
| 100221 | *M. odoratimimus* | 99.5 | 246 | 99.6 | 23 | 31 | 78 | 78 | 5.871 |
| 100223 | *M. odoratimimus* | 99.5 | 246 | 99.6 | 23 | 31 | 78 | 78 | 5.871 |
| 100271 | *M. odoratimimus* | 100.0 | 246 | 100.0 | 23 | 31 | 78 | 78 | 5.871 |
| 100469 | *M. odoratimimus* | 99.6 | 246 | 99.6 | 23 | 31 | 78 | 78 | 5.871 |
| 100471 | *M. odoratimimus* | 95.2 | 246 | 93.9 | 25 | 31 | 78 | 78 | 6.085 |
| 100472 | *M. odoratimimus* | 100.0 | 246 | 100.0 | 23 | 31 | 78 | 78 | 5.871 |
| 100473 | *M. odoratimimus* | 94.8 | 246 | 93.9 | 25 | 32 | 77 | 78 | 5.919 |
| 100474 | *M. odoratimimus* | 100.0 | 246 | 100.0 | 23 | 31 | 78 | 78 | 5.871 |
| 100475 | *M. odoratimimus* | 99.6 | 246 | 99.6 | 23 | 31 | 78 | 78 | 5.871 |
| 100476 | *M. odoratimimus* | 94.8 | 246 | 93.9 | 25 | 32 | 77 | 78 | 5.919 |
| 100477 | *M. odoratimimus* | 99.6 | 246 | 99.6 | 23 | 31 | 78 | 78 | 5.871 |
| 100677 | *M. odoratimimus* | 100.0 | 246 | 100.0 | 23 | 31 | 78 | 78 | 5.871 |
| 100679 | *M. odoratimimus* | 99.5 | 246 | 99.6 | 23 | 31 | 78 | 78 | 5.871 |
| 100682 | *M. odoratimimus* | 99.5 | 246 | 99.6 | 23 | 31 | 78 | 78 | 5.871 |
| 100683 | *M. odoratimimus* | 100.0 | 246 | 100.0 | 23 | 31 | 78 | 78 | 5.871 |
| 100819 | *M. odoratimimus* | 99.5 | 246 | 99.6 | 23 | 31 | 78 | 78 | 5.871 |
| 100820 | *M. odoratimimus* | 94.8 | 246 | 93.9 | 25 | 32 | 77 | 78 | 5.919 |
| 100821 | *M. odoratimimus* | 94.8 | 246 | 93.9 | 25 | 32 | 77 | 78 | 5.919 |
| 100840 | *M. odoratimimus* | 99.6 | 246 | 99.6 | 23 | 31 | 78 | 78 | 5.871 |
| 100841 | *M. odoratimimus* | 100.0 | 246 | 100.0 | 23 | 31 | 78 | 78 | 5.871 |
| 100843 | *M. odoratimimus* | 100.0 | 246 | 100.0 | 23 | 31 | 78 | 78 | 5.871 |
| 100844 | *M. odoratimimus* | 97.0 | 246 | 97.2 | 23 | 31 | 78 | 78 | 5.867 |
| 100859 | *M. odoratimimus* | 99.5 | 246 | 99.6 | 23 | 31 | 78 | 78 | 5.871 |
| 100863 | *M. odoratimimus* | 99.6 | 246 | 99.6 | 23 | 31 | 78 | 78 | 5.871 |
| 100864 | *M. odoratimimus* | 99.5 | 246 | 99.6 | 23 | 31 | 78 | 78 | 5.871 |
| 100865 | *M. odoratimimus* | 100.0 | 246 | 100.0 | 23 | 31 | 78 | 78 | 5.871 |
| 100866 | *M. odoratimimus* | 99.7 | 246 | 99.6 | 23 | 30 | 79 | 78 | 6.025 |
| 100867 | *M. odoratimimus* | 99.7 | 246 | 99.6 | 23 | 30 | 79 | 78 | 6.025 |
| 100889 | *M. odoratimimus* | 95.2 | 246 | 96.4 | 25 | 30 | 78 | 78 | 6.250 |
| 100891 | *M. odoratimimus* | 99.6 | 246 | 99.6 | 25 | 31 | 78 | 78 | 6.085 |
| 100893 | *M. odoratimimus* | 94.9 | 246 | 95.1 | 25 | 30 | 79 | 77 | 6.250 |
| 100894 | *M. odoratimimus* | 100.0 | 246 | 100.0 | 23 | 31 | 78 | 78 | 5.871 |
| 100895 | *M. odoratimimus* | 95.2 | 261 | 96.3 | 26 | 30 | 83 | 85 | 6.423 |
| 100896 | *M. odoratimimus* | 100.0 | 246 | 100.0 | 23 | 31 | 78 | 78 | 5.871 |
| 100897 | *M. odoratimimus* | 95.2 | 246 | 96.4 | 25 | 30 | 78 | 78 | 6.250 |
| 100898 | *M. odoratimimus* | 100.0 | 246 | 100.0 | 23 | 31 | 78 | 78 | 5.871 |
| 100899 | *M. odoratimimus* | 99.6 | 246 | 99.6 | 23 | 31 | 78 | 78 | 5.871 |
| 100920 | *M. odoratimimus* | 94.8 | 246 | 93.9 | 25 | 32 | 77 | 78 | 5.919 |
| 101069 | *M. odoratimimus* | 99.6 | 246 | 99.6 | 23 | 31 | 78 | 78 | 5.871 |
| 101503 | *M. odoratimimus* | 100.0 | 246 | 100.0 | 23 | 31 | 78 | 78 | 5.871 |
| 101504 | *M. odoratimimus* | 100.0 | 246 | 100.0 | 23 | 31 | 78 | 78 | 5.871 |
| 101506 | *M. odoratimimus* | 94.7 | 246 | 93.5 | 25 | 31 | 77 | 79 | 6.085 |
| 101507 | *M. odoratimimus* | 99.5 | 246 | 99.6 | 23 | 31 | 78 | 78 | 5.871 |
| 100222 | *M. odoratus* | 98.5 | 248 | 98.8 | 27 | 29 | 87 | 70 | 6.887 |
| 100681 | *M. odoratus* | 94.9 | 248 | 97.2 | 27 | 29 | 86 | 72 | 6.835 |
| 100818 | *M. odoratus* | 98.5 | 248 | 99.6 | 27 | 29 | 87 | 70 | 6.887 |
| 100842 | *M. odoratus* | 99.3 | 248 | 99.6 | 27 | 29 | 87 | 71 | 6.835 |
| 100919 | *M. odoratus* | 97.1 | 248 | 98.4 | 27 | 31 | 85 | 71 | 6.427 |

Table S6: Strongly basic amino acids (AA): Lysine (K), arginine (R); Strongly acidic amino acids: Asparagine (D), glutamine (E); Hydrophobic amino acids: Alanine (A), isoleucine (I), leucine (L), phenylalanine (F), tryptophan (W), valine (V); Polar amino acids: asparagine (N), cysteine (C), glutamine (Q), serine (S), threonine (T), tyrosine (Y).
