# Supplementary material for: Whole-genome sequencing of a large collection of Myroides odoratimimus and Myroides odoratus isolates and antimicrobial susceptibility studies
Source: Emerg Microbes Infect. 2018 Apr 4;7:61. doi: 10.1038/s41426-018-0061-x (PMC5884818; doi:10.1038/s41426-018-0061-x)
Supplement: Supplementary file 5 — Table S5(DOCX 27 kb) [file 41426_2018_61_MOESM5_ESM.docx]

Table S5: Infection sites and clinical sample types.

| DSM number | Species | Infection site | Clinical sample type | Origin of sample |
| --- | --- | --- | --- | --- |
| 100221 | *M. odoratimimus* | Ulcer, not further specified | Swab | Human |
| 100223 | *M. odoratimimus* | Ulcer, not further specified | Swab | Human |
| 100271 | *M. odoratimimus* | Leg ulcer, interdigital space, left foot | Swab | Human |
| 100469 | *M. odoratimimus* | Chronic wound, right lower leg | Swab | Human |
| 100471 | *M. odoratimimus* | Left big toe | Swab | Human |
| 100472 | *M. odoratimimus* | Erysipelas, erosion, left lower leg | Swab | Human |
| 100473 | *M. odoratimimus* | Left forefoot, leg ulcer | Swab | Human |
| 100474 | *M. odoratimimus* | Left lower leg | Swab | Human |
| 100475 | *M. odoratimimus* | Ulcer, forefoot, not further specified | Swab | Human |
| 100476 | *M. odoratimimus* | Leg ulcer, right lower leg | Swab | Human |
| 100477 | *M. odoratimimus* | Genitourinary tract | Urine | Human |
| 100677 | *M. odoratimimus* | Genitourinary tract | Urine | Human |
| 100679 | *M. odoratimimus* | Not specified | Swab | Human |
| 100682 | *M. odoratimimus* | Leg ulcer, not further specified | Swab | Human |
| 100683 | *M. odoratimimus* | Leg ulcer, not further specified | Swab | Human |
| 100819 | *M. odoratimimus* | Genitourinary tract | Urine | Human |
| 100820 | *M. odoratimimus* | Right lower leg, not further specified | Swab | Human |
| 100821 | *M. odoratimimus* | Lower leg, not further specified | Swab | Human |
| 100840 | *M. odoratimimus* | not specified | Swab | Human |
| 100841 | *M. odoratimimus* | Leg Ulcer, left lower leg | Swab | Human |
| 100843 | *M. odoratimimus* | Right lower leg | Swab | Human |
| 100844 | *M. odoratimimus* | Leg ulcer, left leg | Swab | Human |
| 100859 | *M. odoratimimus* | not specified | Swab | Dog |
| 100863 | *M. odoratimimus* | Ulcer, not further specified | Swab | Human |
| 100864 | *M. odoratimimus* | Not specified | Swab | Horse |
| 100865 | *M. odoratimimus* | Not specified | Swab | Human |
| 100866 | *M. odoratimimus* | Not specified | Swab | Human |
| 100867 | *M. odoratimimus* | Not specified | Swab | Human |
| 100889 | *M. odoratimimus* | Foot, not further specified | Swab | Human |
| 100891 | *M. odoratimimus* | Genitourinary tract | Urine | Human |
| 100893 | *M. odoratimimus* | Not specified | Swab | Human |
| 100894 | *M. odoratimimus* | Not specified | Swab | Human |
| 100895 | *M. odoratimimus* | Not specified | Swab | Human |
| 100896 | *M. odoratimimus* | Not specified | Swab | Human |
| 100897 | *M. odoratimimus* | Not specified | Swab | Human |
| 100898 | *M. odoratimimus* | Not specified | Swab | Human |
| 100899 | *M. odoratimimus* | Not specified | Swab | Human |
| 100920 | *M. odoratimimus* | Leg ulcer, left lower leg | Swab | Human |
| 101069 | *M. odoratimimus* | Left lower leg | Swab | Human |
| 101503 | *M. odoratimimus* | Right foot | Swab | Human |
| 101504 | *M. odoratimimus* | Ulcer, not further specified | Swab | Human |
| 101506 | *M. odoratimimus* | Ulcer, not further specified | Swab | Human |
| 101507 | *M. odoratimimus* | Genitourinary tract | Urine | Human |
| 100222 | *M. odoratus* | Not specified | Swab | Human |
| 100470 | *M. odoratus* | Chronic wound, right lower leg | Swab | Human |
| 100678 | *M. odoratus* | Not specified | Swab | Human |
| 100680 | *M. odoratus* | Not specified | Swab | Human |
| 100681 | *M. odoratus* | Not specified | Swab | Human |
| 100817 | *M. odoratus* | Not specified | Swab | Human |
| 100818 | *M. odoratus* | Not specified | Swab | Human |
| 100839 | *M. odoratus* | Leg ulcer, left leg | Swab | Human |
| 100842 | *M. odoratus* | Leg ulcer, right lower leg | Swab | Human |
| 100857 | *M. odoratus* | Not specified | Swab | Dog |
| 100858 | *M. odoratus* | Not specified | Swab | Greek tortoise |
| 100860 | *M. odoratus* | Not specified | Swab | Human |
| 100861 | *M. odoratus* | Ulcer, not further specified | Swab | Human |
| 100862 | *M. odoratus* | Not specified | Swab | Horse |
| 100890 | *M. odoratus* | Genitourinary tract | Urine | Human |
| 100919 | *M. odoratus* | Erysipelas, right lower leg | Swab | Human |
