# Supplementary material for: Whole-genome sequencing of a large collection of Myroides odoratimimus and Myroides odoratus isolates and antimicrobial susceptibility studies
Source: Emerg Microbes Infect. 2018 Apr 4;7:61. doi: 10.1038/s41426-018-0061-x (PMC5884818; doi:10.1038/s41426-018-0061-x)
Supplement: Supplementary file 6 — Table S6 (DOCX 34 kb) [file 41426_2018_61_MOESM6_ESM.docx]

Table S6: Antibiotics used and MIC ranges covered.

| Antibiotic | MIC range μg/ml |
| --- | --- |
| Ampicillin | 0.016 – 256 |
| Piperacillin/tazobactam | 0.016 – 256 |
| Ceftazidime | 0.016 – 256 |
| Cefepime | 0.016 – 256 |
| Aztreonam | 0.016 – 256 |
| Imipenem | 0.002 – 32 |
| Meropenem | 0.002 – 32 |
| Ciprofloxacin | 0.002 – 32 |
| Levofloxacin | 0.002 – 32 |
| Moxifloxacin | 0.002 – 32 |
| Trimethoprim/sulfamethoxazole | 0.002 – 32 |
| Tigecycline | 0.016 – 256 |
| Fosfomycin | 0.064 – 1024 |
| Colistin | 0.016 – 256 |
| Gentamicin | 0.064 – 1024 |
| Amikacin | 0.016 – 256 |
| Erythromycin | 0.016 – 256 |
| Azithromycin | 0.016 – 256 |
| Daptomycin | 0.016 – 256 |
| Rifampicin | 0.002 - 32 |
