# Supplementary figures and images for: Whole-genome sequencing of a large collection of Myroides odoratimimus and Myroides odoratus isolates and antimicrobial susceptibility studies
Source: Emerg Microbes Infect. 2018 Apr 4;7:61. doi: 10.1038/s41426-018-0061-x (PMC5884818; doi:10.1038/s41426-018-0061-x)

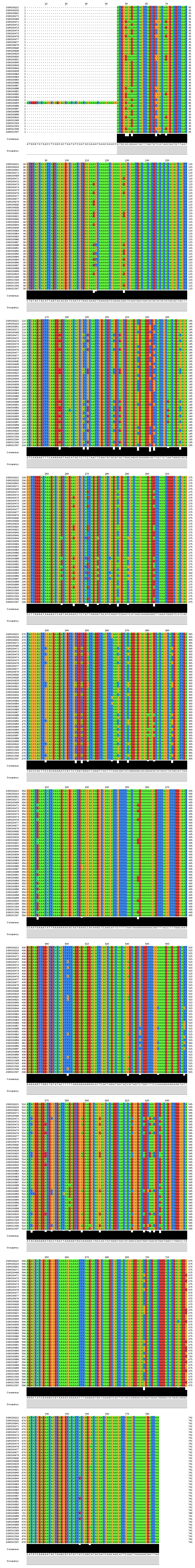

Supplement: Supplementary file 7 — Figure S1(PNG 767 kb) [file 41426_2018_61_MOESM7_ESM.png]

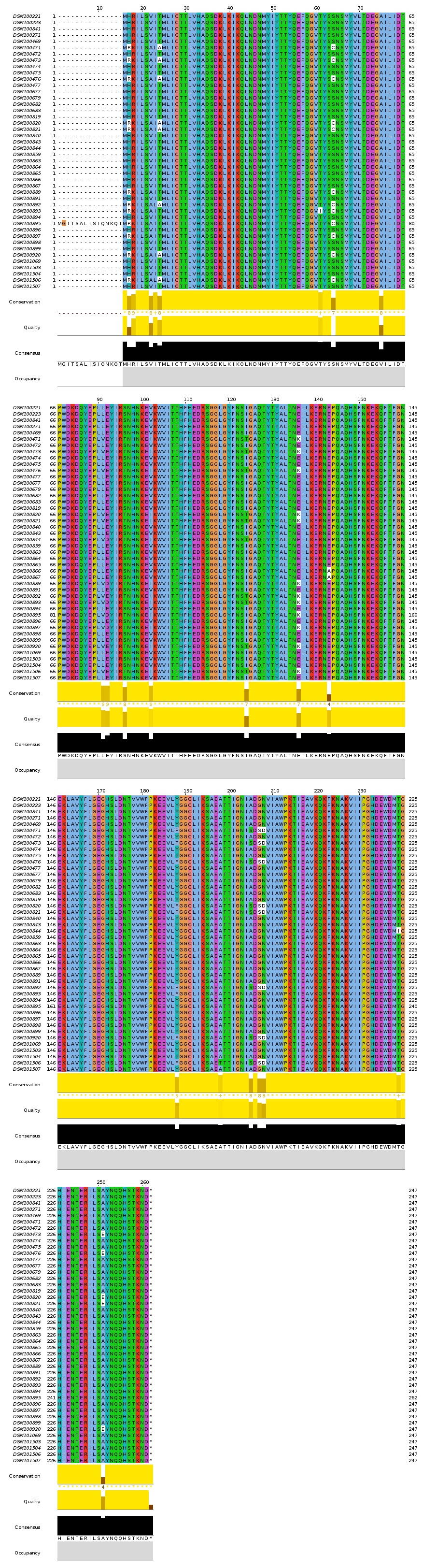

Supplement: Supplementary file 8 — Figure S2(PNG 479 kb) [file 41426_2018_61_MOESM8_ESM.png]

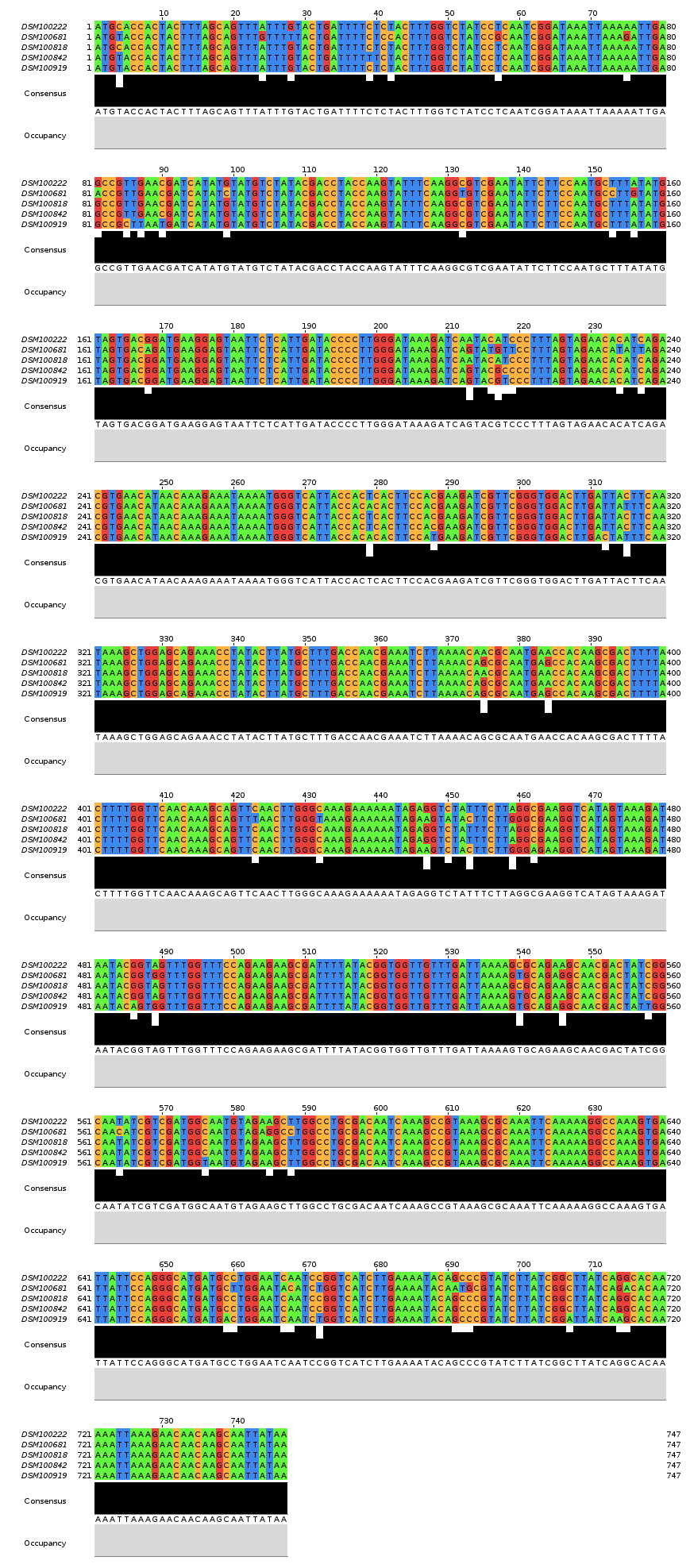

Supplement: Supplementary file 9 — Figure S3(PNG 118 kb) [file 41426_2018_61_MOESM9_ESM.png]

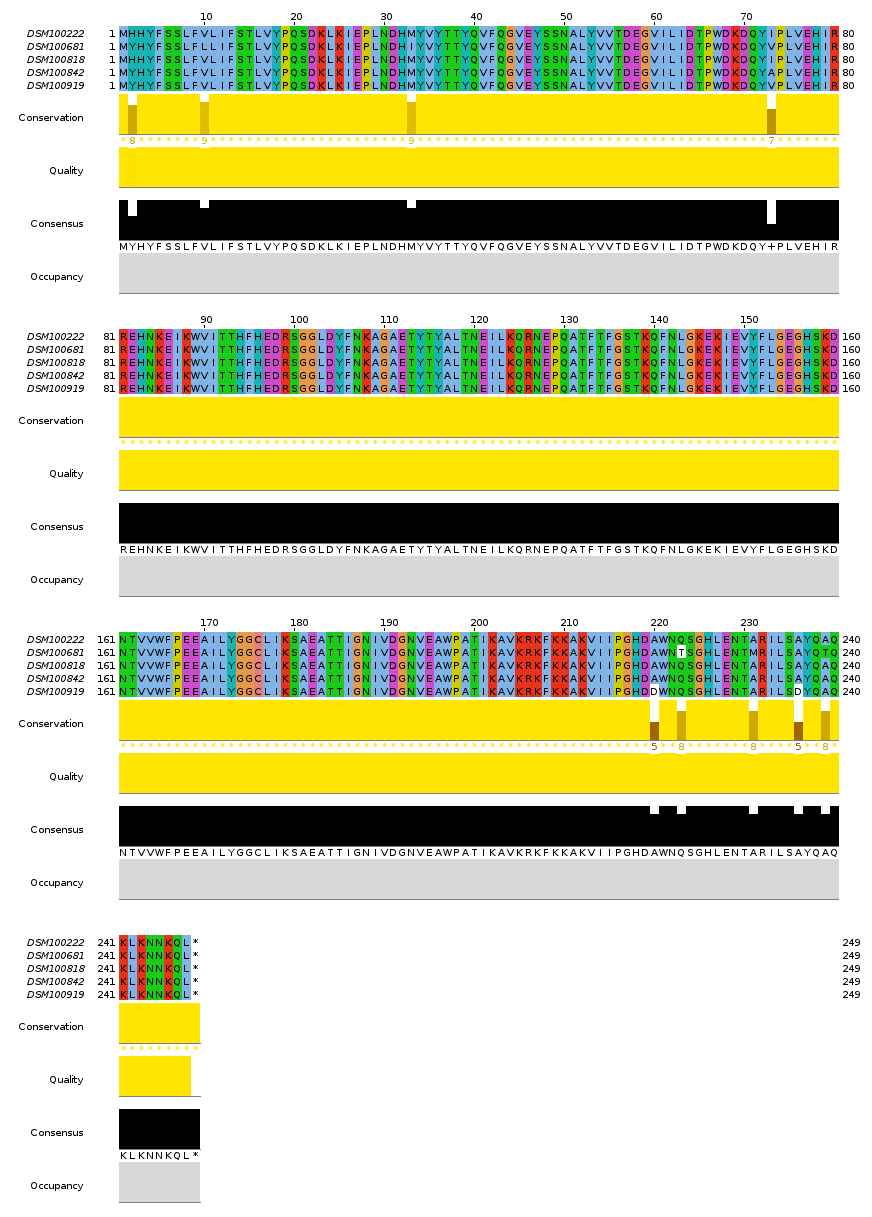

Supplement: Supplementary file 10 — Figure S4(PNG 74 kb) [file 41426_2018_61_MOESM10_ESM.png]
